# Editorial: Chronic subdural hematoma: Overview of recent therapeutic advancements

**DOI:** 10.3389/fneur.2023.1155680

**Published:** 2023-04-03

**Authors:** Homa Sadeghian, Kohei Chida, Rouzbeh Motiei-Langroudi

**Affiliations:** ^1^Department of Neurology, University of Kentucky, Lexington, KY, United States; ^2^Department of Neurosurgery, University of Kentucky, Lexington, KY, United States; ^3^Department of Neurosurgery, Iwate Medical University, Morioka, Iwate, Japan

**Keywords:** chronic subdural hematoma, recurrence, treatment, surgery, pathophysiology

Chronic subdural hematoma (CSDH) is a common form of intracranial hemorrhage. With the aging of population-which increases susceptibility to the disease-it is of utmost importance to expand our understanding of the condition and update treatment protocols. Most cases can be managed conservatively, but in those needing surgery, burr hole craniostomy (BHC) remains the most commonly used technique with favorable outcome; craniotomy and hematoma evacuation are reserved for recurrence (1). Given the older age of the affected population and higher prevalence of co-morbidities, more minimally invasive surgery (MIS) options have been proposed, especially in octogenarians. For instance, twist-drill craniostomy (TDC) is reported to have similar efficacy to BHC with a lower complication rate and length of hospital stay (Wei et al.). However, another review showed that while complications, recurrence, cure, and mortality rates were not different between TDC and BHC, TDC was associated with a higher reoperation rate than BHD, especially if not used with a negative suction drainage system (2). In general, superiority of one technique over the other cannot be conferred from the current data and both are efficient and relatively safe tools. Another MIS option is Subdural Evacuating Port System (SEPS). In the proper setting, SEPS can be done outside operating room with comparable complications, mortality, and recurrence rates (Liu et al.). There are also some promising early results with active bone hole drainage systems, as they reduce post-operative pneumatosis (a known recurrence risk factor) (Zhang et al.).

Regardless of the technique, high recurrence and reoperation rates (10-25%) still pose a unique challenge (3). Among many, anticoagulant use, presence of mixed hematoma signal densities/intensities on CT/MRI, laminar and separated hematomas, larger hematoma residual post-surgery, and severe brain atrophy are some of the known risk factors for recurrence and reoperation (3) (Tian et al.). In this regard, < 50% decrease in hematoma thickness on post-operative CT and < 40% brain re-expansion rate on post-operative day 7-9 are associated with higher recurrence rate (3) (Han et al.).

To tailor treatment paradigms to get better results, we have to understand the pathophysiology better. Historically, the pathophysiology of CSDH formation is believed to be a result of several subclinical hemorrhagic events due to trauma to bridging veins, especially in patients with brain atrophy. While this is still true, several studies have shown that inflammation plays a significant role. Vascular endothelial growth factor (VEGF), fibroblast growth factor (FGF), matrix metalloproteinase (MMP)-9, several interleukins (including IL-6,−8, and−10), and cytokines have been identified as relevant factors in the development of CSDH. The cascade of inflammation, fibrinolysis, and angiogenesis results in formation of fragile vessels inside the CSDH membranes, more micro- or macro-bleeding, and hematoma expansion (4). Several studies have tried to use inflammatory biomarkers obtained from systemic blood or subdural cavity to predict recurrence. For instance, eosinophil-rich peripheral blood (eosinophil counts ≥ 100/μL) and postoperative neutrophil to lymphocyte ratio ≥ 1 (a post-operative ratio that does not decrease compared to the preoperative one) were reported to be independent predictors of CSDH recurrence (4, 5). Another novel marker is red blood cell distribution width (RDW) to platelet count ratio (RPR) (Güresir et al.). Values ≥ 0.0568 have been shown to predict recurrence. These biomarkers are easy to get and provide invaluable information to better predict prognosis and tailor our treatment by identifying patients who might benefit from anti-inflammatory therapy. Dexamethasone (especially if used in conjunction with atorvastatin) has been shown promising in the treatment of CSDH and suppressing the inflammation cascade might be the underlying mechanism (6, 7). However, considering the age and morbidity of CSDH patients, it should be administered with caution (8). Some other recently explored medications with promising results are atorvastatin and tranexamic acid (7). As promising as the early results are, we need more data to support their role in the management of CSDH, either as a primary agent or in conjunction with surgical and interventional procedures. Updating our understanding about the underlying process reveals a need for newer treatments to exclusively target potential culprits, such as VEGF or other potential mediators (Edlmann et al.). Given the role of angiogenesis and VEGF in the disease pathophysiology, an interesting suggestion would be anti-angiogenesis factors, such as bevacizumab. This topic is fresh and not fully investigated yet. A recent study has investigated etiologies else than inflammation cascade (Dubinski et al.). Obtaining swab cultures from subdural space in 44 patients with CSDH recurrence, they noticed a low-grade infection in 29% of the cases, mostly by staphylococcus species. More research is needed to identify (indolent) infection as a potential risk factor for CSDH recurrence, as it can potentially be a target for treatment.

Lastly, a promising intervention that recently has been added to our armamentarium is middle meningeal artery embolization (MMAE). There are several published and ongoing studies investigating MMAE's role in treating both primary and recurrent CSDH, with promising results (9, 10). MMAE not only de-vascularizes the hematoma membrane, resulting in fewer bleeding episodes, but also reduces the influx of inflammatory mediators from the hematoma membrane into the fluid.

Through decades, we have improved our knowledge about pathophysiology of CSDH and added techniques and procedures to our arsenal. Still, there is more we need to know and do in order to give better care to this specific population.

## Author contributions

RM-L is the leader of the Research Topic and HS and KC are Topic Editors. HS and RM-L wrote the draft. All authors contributed to the article and approved the submitted version.
